# Evaluating the Efficacy of Type 2 Diabetes Polygenic Risk Scores in an Independent European Population

**DOI:** 10.3390/ijms25021151

**Published:** 2024-01-17

**Authors:** Monta Brīvība, Ivanna Atava, Raitis Pečulis, Ilze Elbere, Laura Ansone, Maija Rozenberga, Ivars Silamiķelis, Jānis Kloviņš

**Affiliations:** Latvian Biomedical Research and Study Centre, LV-1067 Riga, Latvia; ivanna.atava@biomed.lu.lv (I.A.); ilze.elbere@biomed.lu.lv (I.E.); laura.ansone@biomed.lu.lv (L.A.); klovins@biomed.lu.lv (J.K.)

**Keywords:** type 2 diabetes, polygenic risk score, population stratification

## Abstract

Numerous type 2 diabetes (T2D) polygenic risk scores (PGSs) have been developed to predict individuals’ predisposition to the disease. An independent assessment and verification of the best-performing PGS are warranted to allow for a rapid application of developed models. To date, only 3% of T2D PGSs have been evaluated. In this study, we assessed all (n = 102) presently published T2D PGSs in an independent cohort of 3718 individuals, which has not been included in the construction or fine-tuning of any T2D PGS so far. We further chose the best-performing PGS, assessed its performance across major population principal component analysis (PCA) clusters, and compared it with newly developed population-specific T2D PGS. Our findings revealed that 88% of the published PGSs were significantly associated with T2D; however, their performance was lower than what had been previously reported. We found a positive association of PGS improvement over the years (*p*-value = 8.01 × 10^−4^ with PGS002771 currently showing the best discriminatory power (area under the receiver operating characteristic (AUROC) = 0.669) and PGS003443 exhibiting the strongest association PGS003443 (odds ratio (OR) = 1.899). Further investigation revealed no difference in PGS performance across major population PCA clusters and when compared with newly developed population-specific PGS. Our findings revealed a positive trend in T2D PGS performance, consistently identifying high-T2D-risk individuals in an independent European population.

## 1. Introduction

Large-scale multi-cohort genome-wide association studies (GWAS) meta-analyses have allowed for the construction of polygenic risk scores (PGS) with increasingly higher performance for the prediction of individuals’ genetic liability to phenotype [1]. Many PGS models have been accumulated in the PGS Catalog repository, allowing for a rapid application of new genetic risk scores [1]. However, highly variable model validation approaches, especially the ancestry of validation samples, do not allow for a reliable direct comparison of model efficacy [1]. Furthermore, to assess the applicability of PGS in a heterogeneous population, further assessment in principal component analysis (PCA) stratified groups may be necessary [2]. Finally, if published PGS models are not sufficient for the target application, fine-tuning a new population-specific PGS using established GWAS meta-analyses can further improve disease risk prediction [3,4]. 

As of now, the PGS Catalog has compiled 102 type 2 diabetes (T2D) PGSs from 26 studies. Larger GWAS analyses tend to produce more robust and accurate disease-associated variant effect weights; thus, the majority of developed models employ increasingly larger multi-cohort meta-analyses produced by dedicated consortiums such as DIAGRAM [5] and DIAMANTE [6]. Both meta-analyses and an independent GWAS for weight estimation often involve national biobanks, most notably the biobank study of the United Kingdom (UKB) [7], a large-scale biomedical database of genetics and health information. For summary statistics variable selection and posterior weight estimation, a study-specific genotype-level target dataset is often employed. Fine-tuning over T2D cases and controls from the study population then allows for finding better-performing models, notably for genetically distant target populations that were not included in the original GWAS weight estimation [8]. Recent advances in PGS development methods, however, show that automatically estimated weights can produce results similar to fine-tuning [9,10].

The choice of the PGS construction method also has a crucial effect on model applicability. Current approaches can be distinguished by their prior assumption for variant effect weight distribution. The most common and simplest approach of linkage disequilibrium (LD) pruning + *p*-value thresholding (P + T) assumes zero effect for variants with a *p*-value above a set threshold and no shrinkage. In contrast, more sophisticated Bayesian methods assume no hard threshold for variant exclusion and adjust variant weights based on a set prior and local LD patterns. The LDpred method [10] assumes point normal prior distribution, setting most non-causal effect weights to zero and iterating over the proportion of causal effects. On the other hand, a more recently developed method named PRS-CS [9] assumes continuous shrinkage prior and iterates over differing contributions of the tail-end effect weights. Studies have shown T2D to be highly polygenic with many variants contributing to the etiology of the disease. In this context, PRS-CS has been shown to perform better than other methods [9].

Application of differing PGS model construction methods is benefited by validation in an independent population, both outside and within major continental population clusters, such as between countries of different population histories [4,8]. Currently, only 3% of T2D PGSs available in the PGS Catalog have been independently assessed with evaluation metrics deposited in the PGS Catalog [1]. However, no PGS has been investigated outside the target population, with two studies assessing T2D PGSs in separate populations but for a phenotype other than T2D [11,12]. The Genome Database of Latvian Population (LGDB) possesses one of the largest genotyped T2D cohorts in Europe, relative to the population size [13]. Moreover, currently published T2D PGSs have been developed using 31 populations and 75 cohorts [1], and samples from Latvia and LGDB so far have not been included. This presents a unique opportunity to conduct an independent assessment of T2D polygenic risk scores within Europe. Such an evaluation could greatly contribute to understanding the applicability and accuracy of these scores across diverse European populations.

In this study, we evaluate all currently available T2D PGSs deposited in the PGS Catalog (end date for data retrieval: 19 September 2023) using the reporting framework recommended for the evaluation of PGSs [14]. We compare their performance in an independent European cohort previously not included in the weight calculation of base summary statistics or PGS evaluation. We then choose the best-performing PGS and assess its performance in major population clusters within the LGDB cohort. Finally, based on our findings we fine-tuned effect weights from Mahajan et al.’s 2018 study using data from the population of Latvia by applying the PRS-CS method. We demonstrate that 88% of published PGSs are significantly associated with T2D and the best-performing model PGS002771 performs equally well within major population clusters and when compared with population-specific fine-tuned PGS. We recommend further evaluation of PGS applicability for inclusion in individual T2D risk assessments.

## 2. Results

### 2.1. Cohort Characteristics and Genotype-Based Quality Control

The dataset selected from the Genome Database of Latvian Population (LGDB) for our study initially comprised 3990 genotyped samples and 28,200,578 imputed variants, each with an imputation quality score (Rsq) above 0.3. Through our quality control process, several exclusions were made: 42 individuals were removed due to excess heterozygosity, 24 were identified as having second-degree relatives, and 206 samples were classified as outliers based on principal component analysis (PCA). In terms of variant quality, we excluded 1,969,016 variants that exhibited more than two alleles. Additionally, 18,438,822 polymorphisms were excluded for being rare (minor allele frequency (MAF) of less than 1%), and 393 variants were identified as heterozygosity outliers. Following these exclusions, our main analysis dataset comprised 7,792,347 variants and 3718 individuals (Table S1). Within this cohort, 1496 individuals (40.2%) were diagnosed with type 2 diabetes (T2D). The main demographic characteristics of the participants are shown in Table S1. A majority of the participants were female (62.5%), the average age of participants was 51.5 years (standard deviation (SD) = 14.9), and there were significant age differences between the T2D and control groups (*p*-value < 0.001). The prevailing self-reported ethnic background was Latvian, representing 59.4% of the cohort, exhibiting population clustering among other European populations. In addition, all individuals projected within European (EUR) genetic ancestry PCA space with the 1000 Genomes Project (1000G) dataset as the ground truth (Figure S1, Table S1). To construct and validate the polygenic risk score (PGS), we divided the cohort randomly into two groups. The first group, comprising 70% of the samples (2603 individuals), was used for the construction of the PGS model. The remaining 30% (1115 individuals) formed the validation set, maintaining the same proportion of cases to controls as in the original cohort.

### 2.2. Evaluation of Published T2D PGS Models

**Figure 1:**
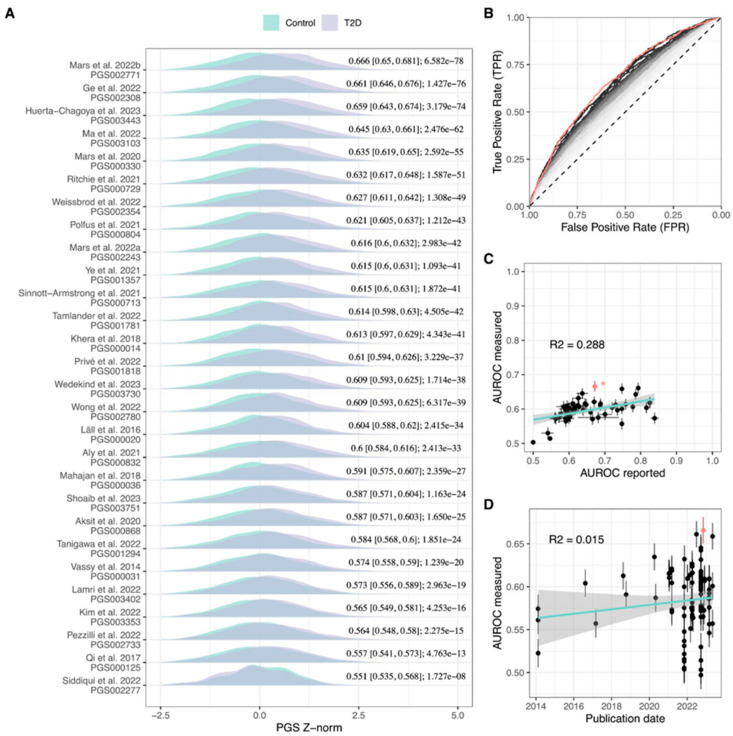
Comparison of T2D PGS performance metrics. (**A**) Distribution of best-performing T2D PGSs for each study included in PGS Catalog and their respective AUROC- and BH-adjusted association *p*-value in the LGDB cohort, (**B**) ROC curves of 102 evaluated T2D PGSs, red line: PGS002771, grey-black gradient: from lowest AUROC PGS (light grey line) increasing to highest AUROC PGS (black line), (**C**) comparison of AUROC reported by the study and measured in the LGDB cohort with asterisk-indicated AUROC predicted from OR, cyan: linear regression line, grey: 95% CI, red dot: AUROC of PGS002771, (**D**) comparison between PGS publishing date and measured AUROC. The highest AUROC PGS002771 is highlighted in red, cyan: linear regression line, grey: 95% CI.AUROC: area under the receiver operating characteristic, ROC: receiver operating characteristic curve, T2D: type 2 diabetes, PGS: polygenic risk score, Z-norm: first 10 principal component-normalized polygenic risk score Z-score. [3,4,6,8,11,15,16,17,18,19,20,21,22,23,24,25,26,27,28,29,30,31,32,33,34,35,36,37].

In total, 102 T2D polygenic scores were evaluated and 88% showed significant association with T2D status (Bonferroni-adjusted family-wise error rate = 4.9 × 10^−4^). A comparison of the performance of evaluated models is depicted in Figure 1, Tables S2–S4, with characteristics of the 10 best-performing PGSs summarized in Table 1. PGS002771 reached the highest area under the receiver operating characteristic (AUROC) value of 0.669 (95% confidence interval (CI) = 0.651–0.686) and explained 10.8% of T2D variance (Negelkarke’s R^2^). The classification performance of PGS002771 demonstrated a notable improvement, reaching an AUROC of 0.757 (95% CI = 0.74–0.773) when accounting for conventional T2D risk factors such as body mass index (BMI), sex, and age. Additionally, a significant difference in performance was observed between the PGS002771 adjusted for conventional risk factors and the model incorporating only the conventional T2D risk factors, reflected as a delta AUROC of 0.046. PGS003443 achieved the highest risk over the SD increase (odds ratio (OR) = 1.89, 95% CI = 1.76–2.05). We also conducted a comparison of the proportion of variants that overlap among the top 10 PGSs with the highest AUROC values (Table S5). The overlap percentage for the highest AUROC PGS002771 was notably low, ranging from 0.01% (with PGS002720) to 0.63% (with PGS003103 and PGS003118). In contrast, other models exhibited higher overlap, with the PGS003443 model, having the highest OR or SD, showing the most overlap with PGS003103 (85.09%) and the least with PGS000729 (8.15%), excluding PGS002771 (0.63% overlap). AUROC evaluation metrics measured in our study were lower compared with the reported (mean AUROC for the values measured in the current study = 0.597, SD = 0.033; mean AUROC for the values reported in the original study = 0.693, SD = 0.068; *p*-value = 1.18 × 10^−6^) with significant correlation between the two measures (r = 0.553, *p*-value = 7.94 × 10^−6^). When reported AUROC values above 0.65 were excluded, the correlation increased to r = 0.895 (Figure 1). There was no significant association between the year of PGS development and measured AUROC (*p*-value = 0.103); however, incremental yearly improvement of PGS performance becomes evident when selecting the ten highest AUROC polygenic scores for each year (*p*-value = 8.01 × 10^−4^) (Figure 1).

Investigation of non-genetic predictors alone revealed a comparably high classification ability with AUROC of 0.711 (95% CI = 0.693–0.728); however, it was significantly lower when evaluated in separate age groups (comparison between AUROC of model comprising non-genetic covariates in the whole LGDB cohort and AUROC of the same model in the second age tertile group solely: D = 6.018, df = 1846, *p*-value = 2.1 × 10^−9^) with lowest AUROC values in the first (AUROC = 0.538, 95% CI = 0.497–0.578) and third age tertile (AUROC = 0.558, 95% CI = 0.522 = 0.593) (Table S4). The inclusion of PGS significantly improved T2D classification in all age groups (comparison between AUROC of model comprising non-genetic covariates and AUROC of PGS002771 adjusted for non-genetic covariates: Z = −3.3217, *p*-value = 8.94 × 10^−4^) with the highest delta gain in the third age tertile when compared with non-genetic covariates only (delta of AUROC for model comprising non-genetic covariates only and AUROC of PGS002771 adjusted for non-genetic covariates = 0.146, both calculated for the third age tertile) (Table S4).

The classification performance of PGS002771 was significantly higher than 98% of evaluated PGSs (BH-adjusted *p*-value < 0.05), with comparable AUROC achieved by PGS003443 (BH-adjusted *p*-value = 0.626) and PGS002308 (*p*-value = 0.251) (Table S3). In general, the highest AUROC PGSs were characterized by the inclusion of >1 × 10^6^ variants, with a number of overlapping polymorphisms significantly associated with PGS AUROC (*p*-value = 2.5 × 10^−4^) (Figure S2).

### 2.3. PGS Performance in Ancestry Clusters

**Figure 2:**
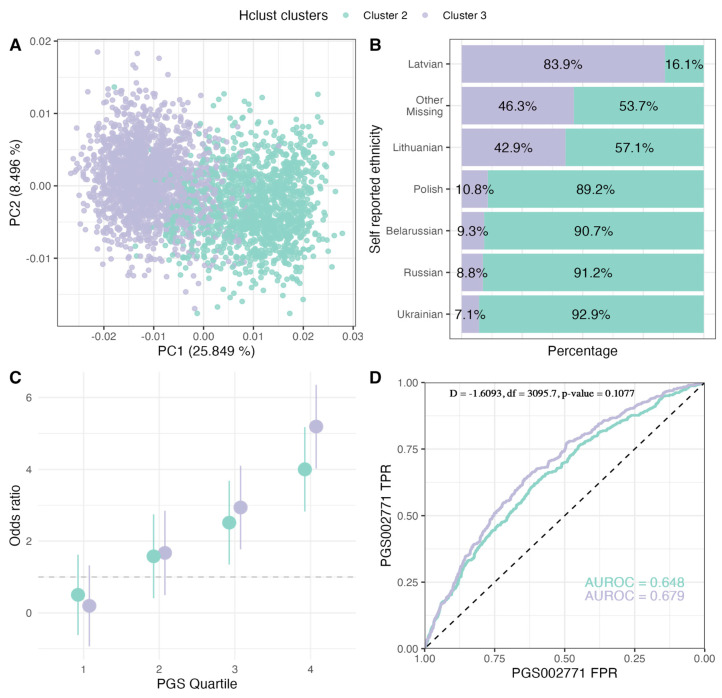
Evaluation of PGS002771 in major population clusters of the LGDB cohort. (**A**) PCA of population clusters, (**B**) proportion of individuals in each cluster depending on their self-assigned ethnicity, (**C**) T2D OR comparison in PGS002771 quartile with the 1st quartile as the reference, (**D**) T2D classification ROC curves comparison between the clusters. LGDB: Genome Database of Latvian Population, PCA: principal component analysis, T2D: type 2 diabetes, OR: odds ratio.

Using the best-performing PGS002771, we further investigated PGS applicability between major ancestries characteristic for the population of Latvia. We first defined two major population groups using hclust hierarchical clustering algorithm. The highest adjusted Rand index (ARI) = 0.413 with a self-assigned ethnicity (SAE) as a ground truth was the eight-cluster model (Figure S3; Table S6), with 89.9% of the population assigned to cluster 3 (N = 1934) and cluster 2 (N = 1411) (Table 2; Figure 2). Cluster 3 was characterized by a majority of Latvian SAE (90.0%) while only 23.6% of the cluster 2 cohort had Latvian SAE. The distribution of different SAEs among these clusters is illustrated in Figure 2. We found that clusters differed significantly in the proportion of T2D cases (X-squared = 111.43, df = 1, *p*-value < 2.2 × 10^−16^) as well as age (W = 1.04 × 10^6^, *p*-value = 1.88 × 10^−9^) and BMI (W = 1.05 × 10^6^, *p*-value = 9.08 × 10^−8^) distributions (Table 2). However, despite the divergence of non-genetic factors between the two clusters, there was no significant difference in PGS002771 classification performance (the comparison between AUROC of cluster 3 and AUROC of cluster 2: D = −1.609, df = 3095.7, *p*-value = 0.108) (Figure 2).

### 2.4. Population-Specific PGS Development

**Figure 3:**
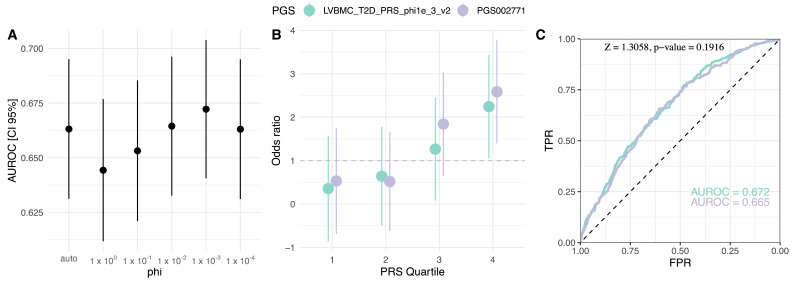
Comparison of the population-specific PGSlvbmc1e_3 and PGS002771. (**A**) Phi global shrinkage parameter effect on the PGS AUROC, (**B**) OR comparison in PGS quartiles with the first quartile as the reference, (**C**) discrimination power comparison ROC curves. PGS: polygenic risk score, AUROC: area under the receiver operating characteristic, OR: odds ratio, TPR: true positive rate, FPR: false positive rate.

Evaluation of published T2D PGSs indicated a common base summary statistics (GWAS catalog ID: GCST009379) and development method PRS-CS (or PRS-CSx) for the best-performing models (Table 1). To test the possible benefits of dataset-specific fine-tuning of PGS, we employed this best practice and constructed six new PGS models over the range of phi parameters (Table S7). The best-developed model LVBMC_T2D_PRS_phi1e_3_v2 (phi = 1 × 10^−3^, N variants = 1,088,653) matched the performance of PGS002771 (Z = 1.3058, *p*-value = 0.1916) and exceeded AUROC of published PGS both for the base model (AUROC = 0.672, 95% CI = 0.641, 0.704) and adjusted for T2D covariates (AUROC = 0.754, 95% CI = 0.725–0.784), explaining 11.7% of T2D variation (Negelkerke’s R^2^) (Figure 3; Table S7). Other constructed PGSs performed equally well (BH adjusted *p*-value > 0.05) except for phi = 1 (Z = 2.7983, *p*-value = 5.13 × 10^−3^) (Table S7). When the top decile was compared against the bottom 90% of the cohort, PGSlvbmc1e_3 reached OR of 2.68 (95% CI = 1.79–4.02, *p*-value (Wald’s test) < 0.00)], increasing further to OR = 4.23 (95% CI = 2.31–7.75, *p*-value < 0.001) when comparing the highest 5% PGS subgroup to the bottom 95%. We also investigated T2D case fraction within outer quartiles, where the comparison between PGSlvbmc1e_3 and PGS002771 showed similar proportions with no significant difference between the two (first quartile: X-squared = 0.547, df = 1, *p*-value = 0.459; fourth quartile: X-squared = 0.118, df = 1, *p*-value = 0.731) (Figure S4).

## 3. Discussion

Before applying the risk prediction models, it is important to evaluate and calibrate the magnitude of the association of common genetic variants in the studies that are independent of the original genome-wide association studies (GWAS). In this study, we performed a comprehensive, independent assessment of all 102 currently published type 2 diabetes (T2D) polygenic risk scores (PGSs), using the genotypes and phenotypic data of the Latvian population from the Genome Database of Latvian Population (LGDB) dataset. Our sample consisted of 3718 genotyped individuals, representing a broad cross-section of the Latvian population. We discovered that 88% of these PGSs showed a significant association with T2D in our population, indicating the overall performance robustness of developed PGSs across different populations at least within Europe. However, the measured performance of tested PGSs was generally lower than reported by their respective source study. This difference might be explained by different study populations’ genetic backgrounds, covariates included, and the limited size of the sample population used in this study, confirming the need for a standardized approach in PGS reporting and assessment [4,14]. Notably, our analysis highlighted PGS002771, which outperformed 98% of the other scores. It achieved an area under the receiver operating characteristic (AUROC) of 0.669, demonstrating equal effectiveness in major population clusters, and was comparable with newly developed, fine-tuned, population-specific PGSs. Importantly, for the majority of models, we observed a strong correlation between the source-reported AUROC values and those obtained in our study (Figure 1C). While some studies have reported unusually high AUROC values for T2D, we were not able to replicate these, most probably indicating an inflated source value. We thus suggest that such a comparison may be used to exclude the models that do not follow the correlation trend for a particular population.

Remarkably, our findings reveal a consistent improvement in T2D PGSs over time, as illustrated in Figure 1D. Due to the larger number of developed PGSs over the last few years, there is also a greater variability in reported AUROC values. However, when the top ten scores annually are considered for evaluation, this correlation becomes highly significant. This positive trajectory in PGS performance coincides with the development of the method, with the first Bayesian regression-based T2D model published in 2018 [24] and the first PGS with continuous shrinkage priors published in 2022 [23]. It is therefore important to perform a continuous evaluation of new PGS models and regularly update the methodology when implementing these approaches into practice.

Most of the evaluated scores were developed using European ancestry GWAS summary statistics, derived predominantly from large-scale meta-analyses. Notably, DIAGRAM [5] and DIAMANTE [6] served as full or partial sources for variant effect weights in nine of the top ten models. Diverging from other base GWAS, the Mahajan et al. 2018 study, which was used in three of the best-performing PGSs, incorporated pancreatic islet-specific regulatory information into its weight estimates. This approach may explain the enhanced discriminatory power these models exhibited for the T2D phenotype. Conversely, when models based on Mahajan et al., 2018, underperformed [11,28,29], they typically employed non-Bayesian methods, such as pruning and *p*-value thresholding (P + T) or selecting genome-wide significant variants, an approach that has previously been shown to significantly impede PGS performance [4].

Furthermore, all of the top five models employed Bayesian regression and continuous shrinkage priors, implemented in PRS-CS and its advanced version, PRS-CSx [9]. Among these, four of the five models with the highest AUROC scores utilized the PRS-CS ‘auto’ option. The exception was the model described by Huerta-Chagoya et al., 2023, which instead conducted a grid search over the phi parameters [9]. Despite the top three models relying on identical summary statistics and PGS development methods, PGS002771 stood out in our study. This model was constructed using a target set from a geographically adjacent Finnish population [15] enabling a more refined selection of region-specific variants, most likely enhancing its applicability and relevance for the Latvian population.

An essential consideration in the effective application of PGSs is the potential ethnic heterogeneity within the population. Latvia’s demographic structure underwent significant changes due to the massive wave of immigration between 1945 and 1989. To assess how population subgroup-specific variants influence PGS performance, we analyzed its applicability across major population groups. These groups were identified using a hierarchical clustering algorithm. Principal component analysis (PCA) loading-based clustering revealed two primary ancestry clusters in Latvia, distinguished mainly by either Latvian or Slavic-speaking self-assigned ethnicity (SAE) presence. Despite notable differences in non-genetic factors between these clusters and the known susceptibility of PGSs to ancestral or local regional stratification [8,38], our findings indicate that the PGSs performed consistently well across both groups. This is in line with similar results from other studies in highly heterogeneous populations [2]. However, it is important to note that our use of PCA loadings to adjust for population stratification may have reduced the apparent differences between these groups [39]. Nonetheless, this finding supports the broader applicability of PGS, particularly for T2D, across the entire Latvian population.

Building on previous research suggesting potential benefits of population-specific, fine-tuned PGSs [3], we best practiced for this approach by applying them to the three models with the highest T2D discriminatory power: PGS002771, PGS003443, and PGS002308. All these models used the same GWAS association weights source [6], a model construction method incorporating continuous shrinkage prior via PRS-CS, and, except for PGS003443, an automatic global shrinkage parameter phi. Our exploration of a broader range of phi parameters revealed that phi = 1 × 10^−3^ yielded the best performance, suggesting a less polygenic nature of T2D etiology in contrast to previous findings like Huerta-Chagoya et al., 2023 (PGS003443) [17], where phi = 1 × 10^−2^ was identified as the most effective. Although our development of population-specific PGS did not significantly enhance performance metrics, integrating a local linkage disequilibrium (LD) reference and specific GWAS weights could potentially improve results [3,4].

Our study had some weaknesses. Demographic and anthropometric characteristics were missing for some study subjects (2.2–3.3%). Nevertheless, we decided not to exclude these samples to reach the higher power for analysis. In addition, median age was different between cases and controls, and this may have some impact on the PGS performance.

Although most PGSs were strongly associated with T2D status, efficacy for discriminating for high-T2D-risk individuals currently do not reach clinical utility [40] and might even exaggerate health disparities [41]. Further improvements, however, might be achieved by the incorporation of T2D pathway-specific information for weight estimation [6,42] and augmented weight priors tailored specifically for T2D etiology [9,10].

## 4. Methods

### 4.1. Cohort Description and Data Selection

This study utilized a total of 1630 patients with type 2 diabetes (T2D) and 2360 control subjects, all selected from the Genome Database of the Latvian Population (LGDB) [13]. The T2D group comprised participants who had a clinical diagnosis of T2D, as indicated by the International Classification of Diseases-10 (ICD-10) code E11, and for whom genotype data were available. For the control group, we applied two key exclusion criteria: (1) we excluded any LGDB participant who had a clinically reported or self-reported diagnosis of diabetes (codes E10–E14 in the ICD-10 classification) and (2) those with a history of using antidiabetic treatments. In addition to the genotype data, we collected information on anthropometric measures and self-reported ethnicity at the time of each participant’s enrolment in the LGDB. Written broad consent was obtained from every subject during the recruitment in LGDB (Approval by Central Medical Ethics Committee No. 01-29.1.2/6407). This study was conducted in accordance with the Declaration of Helsinki. The study protocol was approved by the Central Medical Ethics Committee of Latvia (Approval No. 01-29.1/2223).

### 4.2. Genotype Quality Control

Selected genotypes consisted of six batches genotyped with the Infinium Global Screening Array (Illumina, California, USA) on the iScan System microarray scanner (Illumina, USA) from 2016 to 2022 with 192 to 768 samples per batch. Each batch underwent quality control and harmonization, with the merged set resulting in 3990 individuals (1630 cases and 2360 controls) and a total number of 115,362 variants across autosomes with genotype missingness of <0.01. LGDB samples were subsequently imputed with the GRCh38 TOPMed R2 1.0.0 [43] imputation panel using the Michigan imputation server and variants were selected with imputation quality score Rsq > 0.3.

For post-imputation quality, control parameters recommended for polygenic risk score (PGS) analysis were used [44,45]. Variants were selected with genotyping rate > 0.99, heterozygosity P > 1 × 10 × 10^6^, and minor allele frequency (MAF) > 1%. We did not remove variants with an allele count below 100 in both control and case groups as external linkage disequilibrium (LD) reference of 1000G European ancestry individuals was used in this study [44]. Sample-wise, individuals were excluded if missingness exceeded > 0.05, and heterozygosity was calculated outside three standard deviations (SD) of PLINK–het and pi_hat above the 0.1875 threshold set for second-degree relatives. For filtering steps, we used PLINK [46] and Bcftools [47].

### 4.3. Polygenic Risk Score Calculation

To calculate T2D PGSs available in PGScatalog, genotype dosages were used in PGScatalog/pgsc_calc v2.0.0-alpha.3 nextflow pipeline [1]. To correct for the impact of population stratification by addressing differences in mean and variance of PGSs among genetic ancestry groups and identifying population structure based on sample principal component analysis (PCA) loadings, the parameter -run-ancestry was set. The chosen approach adjusts PGS using the first 10 PCA loadings and normalizes the score based on standard deviation in the merged 1000G [48] and Human Genomes Project (HGP) reference population [49].

### 4.4. PGS Evaluation

To evaluate the performance of 102 available T2D PGSs, standard quality metrics for each score were calculated using R (v4.2.1, Vienna, Austria) [50]. The area under the receiver operator characteristic curve (AUC) was assessed using a pROC v1.18.5 package [51], comparing PGS values with LGDB-defined T2D phenotype. R package psych v2.3.9 [52] was used for correlation coefficient calculation while epiDisplay v3.5.0.2 [53] allowed us to extract odds ratio (OR) over SD increase from the generalized linear model (glm) result. These metrics were subsequently compared with corresponding values reported by the PGS source study. As the AUC value was not reported by PGS002771, we predicted PGS AUC from the reported OR, given a 0.962 correlation between AUC and OR values in other included PGS. For stratified evaluation among age groups, the cohort was divided into tertiles (first tertile: minimum age = 5–maximum age = 47, second: 47–59, third: 59–94).

Additionally, for the best-performing PGS, separate evaluations of its performance in major PCA clusters were assessed. Clusters were formed based on Euclidean distance between the first 10 principal components using hierarchical clusterization implemented in R function hclust [50]. A number of optimal clusters were selected manually based on the evaluation of clusterization accuracy by adjusted rank index (ARI) as implemented in R package mclust v6.0.1 [54]. Data on participants’ self-assigned ethnicity was used as ground truth labels.

We used multiple methods to assess the relationships between genetic and non-genetic factors in relation to T2D status. For non-parametric tests, we used a two-sample Wilcoxon test while significant differences between receiver operator characteristic (ROC) curves were assessed using roc.test function [51]. Association with T2D status was tested using a generalized linear model for binomial outcomes as implemented in the glm function, with further calculating Negelkarke’s R2, a pseudo-R2 statistic as implemented in the fmsb v0.7.5 package [55]. The *p*-value was adjusted according to the Benjamini–Hochberg (BH) procedure by applying the p.adjust function on the glm-produced vector of *p*-values in R [50]. Family-wise error rate was corrected using the Bonferroni method dividing 0.05 by the number of evaluated PGS (n = 102). Trendlines for Figure 1C,D were produced with R ggplot::geom_smooth (method = ‘lm’) function [50].

### 4.5. Polygenic Risk Score Model Construction

To develop a polygenic risk score fine-tuned for the LGDB cohort, the PRS-CS v1.1.0 Bayesian regression method with continuous shrinkage prior was used [9]. To maximize scores performance, manually adjusted multiple phi global shrinkage parameters were chosen by a small-scale grid search over phi = 1 × 10^−4^, 1 × 10^−3^, 1 × 10^−2^, 1 × 10^−1^, and 1, with phi value of 1 × 10^−2^ recommended for highly polygenic traits such as type 2 diabetes [9]. For an independent linkage disequilibrium estimation, a 1000G reference of European ancestry was chosen.

## 5. Conclusions

In conclusion, our study provides the first comprehensive evaluation of T2D PGSs within the Latvian population, revealing the top-performing model, PGS002771, and affirming the general robustness of the majority of assessed PGSs in this previously unstudied European cohort. Notably, our findings underscore a positive trajectory in the continuous improvement of T2D PGSs over time, emphasizing the need for ongoing research and updates to enhance their precision and utility in diverse populations and clinical settings. Applying the best practices from previously developed PGSs, we constructed a population-specific T2D PGS that matched the performance of PGS002771. Finally, our analysis revealed the consistent and effective performance of PGSs across major LGDB population ancestry clusters, supporting the broader applicability of T2D PGS across the entire Latvian population.

## Figures and Tables

**Table 1:** Development methods and performance metrics of selected 10 polygenic risk scores with the highest type 2 diabetes discriminatory power in the cohort of the Latvian population.

PGS Author	GWAS Weight Source Study	PGS Development Method *	PGS Variants (% Match)	AUROC [95% CI]	OR/SD [95% CI]
PGS002771 Mars et al., 2022b [15]	GCST009379	PRS-CS	1,091,608 (97.7)	0.669 [0.651, 0.686]	1.883 [1.748, 2.028]
PGS002308 Ge et al., 2022 [16]	GCST009379	PRS-CSx	1,259,754 (86.9)	0.665 [0.648, 0.683]	1.883 [1.747, 2.031]
PGS003443 Huerta-Chagoya et al., 2023 [17]	GCST009379	PRS-CSx	1,092,496 (98.5)	0.665 [0.648, 0.683]	1.899 [1.759, 2.049]
PGS003103 Ma et al., 2022 [18]	GCST006867	PRS-CS	945,820 (98.9)	0.651 [0.633, 0.668]	1.795 [1.663, 1.937]
PGS003118 Ma et al., 2022 [18]	GCST006867	PRS-CS	945,921 (98.9)	0.649 [0.631, 0.666]	1.783 [1.652, 1.923]
PGS000330 Mars et al., 2020 [19]	GCST009379	LDpred	6,437,380 (87.2)	0.643 [0.625, 0.661]	1.743 [1.616, 1.88]
PGS003099 Ma et al., 2022 [18]	GCST006867	lassosum	555,512 (83.4)	0.636 [0.618, 0.654]	1.724 [1.594, 1.864]
PGS002354 Weissbrod et al., 2022 [4]	Weissbrod et al., 2022 [4]	BOLT-LMM	1,109,311 (97.4)	0.633 [0.615, 0.651]	1.636 [1.523, 1.757]
PGS000729 Ritchie et al., 2021 [20]	GCST007517	LD thinning	2,017,388 (80.9)	0.633 [0.615, 0.651]	1.611 [1.502, 1.728]
PGS002720 Weissbrod et al., 2022 [4]	Weissbrod et al., 2022 [4]	SBayesR	911,809 (98)	0.631 [0.613, 0.649]	1.618 [1.508, 1.736]

PGS: polygenic risk score, GWAS: genome-wide association study, AUROC: area under the receiver operating characteristic, OR: odds ratio, SD: standard deviation; CI: confidence interval. * Please find Table S8 for the description of methods applied for the development of evaluated PGSs.

**Table 2:** Cohort characteristics of two major Hclust clusters based on the first 10 PCs in the population of Latvia.

	Cluster 3			Cluster 2		
	Control.	T2D	All	Control	T2D	All
	(N = 1317)	(N = 617)	(N = 1934)	(N = 705)	(N = 706)	(N = 1411)
Sex						
Female	808 (61.4%)	388 (62.9%)	1196 (61.8%)	444 (63.0%)	450 (63.7%)	894 (63.4%)
Male	469 (35.6%)	217 (35.2%)	686 (35.5%)	237 (33.6%)	249 (35.3%)	486 (34.4%)
Missing	40 (3.0%)	12 (1.9%)	52 (2.7%)	24 (3.4%)	7 (1.0%)	31 (2.2%)
Age						
Mean (SD)	46.9 (14.3)	56.6 (13.6)	50.0 (14.8)	47.3 (14.8)	58.8 (12.4)	53.2 (14.8)
Median [Min, Max]	47.0 [5.00, 94.0]	58.0 [18.0, 85.0]	50.0 [5.00, 94.0]	48.0 [9.00, 88.0]	60.0 [19.0, 89.0]	55.0 [9.00, 89.0]
Missing	40 (3.0%)	12 (1.9%)	52 (2.7%)	28 (4.0%)	7 (1.0%)	35 (2.5%)
BMI						
Mean (SD)	26.6 (5.38)	32.5 (6.72)	28.5 (6.47)	27.3 (7.78)	32.3 (6.36)	29.8 (7.52)
Median [Min, Max]	25.7 [11.3, 51.0]	32.3 [18.2, 56.4]	27.5 [11.3, 56.4]	26.6 [14.7, 163]	31.8 [18.7, 61.3]	29.1 [14.7, 163]
Missing	57 (4.3%)	19 (3.1%)	76 (3.9%)	31 (4.4%)	15 (2.1%)	46 (3.3%)
Ethnicity						
Belarussian	5 (0.4%)	5 (0.8%)	10 (0.5%)	43 (6.1%)	55 (7.8%)	98 (6.9%)
Latvian	1213 (92.1%)	528 (85.6%)	1741 (90.0%)	233 (33.0%)	100 (14.2%)	333 (23.6%)
Lithuanian	11 (0.8%)	7 (1.1%)	18 (0.9%)	14 (2.0%)	10 (1.4%)	24 (1.7%)
Other Missing	53 (4.0%)	29 (4.7%)	82 (4.2%)	47 (6.7%)	48 (6.8%)	95 (6.7%)
Polish	3 (0.2%)	4 (0.6%)	7 (0.4%)	25 (3.5%)	33 (4.7%)	58 (4.1%)
Russian	29 (2.2%)	42 (6.8%)	71 (3.7%)	321 (45.5%)	417 (59.1%)	738 (52.3%)
Ukrainian	3 (0.2%)	2 (0.3%)	5 (0.3%)	22 (3.1%)	43 (6.1%)	65 (4.6%)

T2D: type 2 diabetes patients, SD: standard deviation, Min: minimum value, Max: maximum value.

## Data Availability

All data used in this study can be requested from the Genome Database of the Latvian Population.

## Supplementary Materials

The following supporting information can be downloaded at: https://www.mdpi.com/article/10.3390/ijms25021151/s1. References [9,10,20,56,57,58,59] are cited in Supplementary Material.

## References

[1] Lambert S.A., Gil L., Jupp S., Ritchie S.C., Xu Y., Buniello A., McMahon A., Abraham G., Chapman M., Parkinson H. (2021). The Polygenic Score Catalog as an Open Database for Reproducibility and Systematic Evaluation. Nat. Genet..

[2] Albert E.A., Kondratieva O.A., Baranova E.E., Sagaydak O.V., Belenikin M.S., Zobkova G.Y., Kuznetsova E.S., Deviatkin A.A., Zhurov A.A., Karpulevich E.A. (2023). Transferability of the PRS Estimates for Height and BMI Obtained from the European Ethnic Groups to the Western Russian Populations. Front. Genet..

[3] Läll K., Mägi R., Morris A., Metspalu A., Fischer K. (2017). Personalized Risk Prediction for Type 2 Diabetes: The Potential of Genetic Risk Scores. Genet. Med..

[4] Weissbrod O., Kanai M., Shi H., Gazal S., Peyrot W.J., Khera A.V., Okada Y., Matsuda K., Yamanashi Y., Furukawa Y. (2022). Leveraging Fine-Mapping and Multipopulation Training Data to Improve Cross-Population Polygenic Risk Scores. Nat. Genet..

[5] Scott R.A., Scott L.J., Mägi R., Marullo L., Gaulton K.J., Kaakinen M., Pervjakova N., Pers T.H., Johnson A.D., Eicher J.D. (2017). An Expanded Genome-Wide Association Study of Type 2 Diabetes in Europeans. Diabetes.

[6] Mahajan A., Taliun D., Thurner M., Robertson N.R., Torres J.M., Rayner N.W., Payne A.J., Steinthorsdottir V., Scott R.A., Grarup N. (2018). Fine-Mapping Type 2 Diabetes Loci to Single-Variant Resolution Using High-Density Imputation and Is-let-Specific Epigenome Maps. Nat. Genet..

[7] Bycroft C., Freeman C., Petkova D., Band G., Elliott L.T., Sharp K., Motyer A., Vukcevic D., Delaneau O., O’Connell J. (2018). The UK Biobank Resource with Deep Phenotyping and Genomic Data. Nature.

[8] Privé F., Aschard H., Carmi S., Folkersen L., Hoggart C., O’Reilly P.F., Vilhjálmsson B.J. (2022). Portability of 245 Polygenic Scores When Derived from the UK Biobank and Applied to 9 Ancestry Groups from the Same Cohort. Am. J. Hum. Genet..

[9] Ge T., Chen C.-Y., Ni Y., Feng Y.-C.A., Smoller J.W. (2019). Polygenic Prediction via Bayesian Regression and Continuous Shrinkage Priors. Nat. Commun..

[10] Vilhjalmsson B., Yang J., Finucane H.K., Gusev A., Lindstrom S., Ripke S., Genovese G., Loh P.-R., Bhatia G., Do R. (2015). Modeling Linkage Disequilibrium Increases Accuracy of Polygenic Risk Scores. Am. J. Hum. Genet..

[11] Aksit M.A., Pace R.G., Vecchio-Pagán B., Ling H., Rommens J.M., Boelle P.-Y., Guillot L., Raraigh K.S., Pugh E., Zhang P. (2020). Genetic Modifiers of Cystic Fibrosis-Related Diabetes Have Extensive Overlap With Type 2 Diabetes and Related Traits. J. Clin. Endocrinol. Metab..

[12] Oram R.A., Sharp S.A., Pihoker C., Ferrat L., Imperatore G., Williams A., Redondo M.J., Wagenknecht L., Dolan L.M., Lawrence J.M. (2022). Utility of Diabetes Type–Specific Genetic Risk Scores for the Classification of Diabetes Type Among Multiethnic Youth. Diabetes Care.

[13] Rovite V., Wolff-Sagi Y., Zaharenko L., Nikitina-Zake L., Grens E., Klovins J. (2018). Genome Database of the Latvian Population (LGDB): Design, Goals, and Primary Results. J. Epidemiol..

[14] Wand H., Lambert S.A., Tamburro C., Iacocca M.A., O’Sullivan J.W., Sillari C., Kullo I.J., Rowley R., Dron J.S., Brockman D. (2021). Improving Reporting Standards for Polygenic Scores in Risk Prediction Studies. Nature.

[15] Mars N., Lindbohm J.V., della Briotta Parolo P., Widén E., Kaprio J., Palotie A., Ripatti S. (2022). Systematic Comparison of Family History and Polygenic Risk across 24 Common Diseases. Am. J. Hum. Genet..

[16] Ge T., Irvin M.R., Patki A., Srinivasasainagendra V., Lin Y.-F., Tiwari H.K., Armstrong N.D., Benoit B., Chen C.-Y., Choi K.W. (2022). Development and Validation of a Trans-Ancestry Polygenic Risk Score for Type 2 Diabetes in Diverse Populations. Genome Med..

[17] Huerta-Chagoya A., Schroeder P., Mandla R., Deutsch A.J., Zhu W., Petty L., Yi X., Cole J.B., Udler M.S., Dornbos P. (2023). The Power of TOPMed Imputation for the Discovery of Latino-Enriched Rare Variants Associated with Type 2 Diabetes. Diabetologia.

[18] Ma Y., Patil S., Zhou X., Mukherjee B., Fritsche L.G. (2022). ExPRSweb: An Online Repository with Polygenic Risk Scores for Common Health-Related Exposures. Am. J. Hum. Genet..

[19] Mars N., Koskela J.T., Ripatti P., Kiiskinen T.T.J., Havulinna A.S., Lindbohm J.V., Ahola-Olli A., Kurki M., Karjalainen J., Palta P. (2020). Polygenic and Clinical Risk Scores and Their Impact on Age at Onset and Prediction of Cardiometabolic Diseases and Common Cancers. Nat. Med..

[20] Ritchie S.C., Lambert S.A., Arnold M., Teo S.M., Lim S., Scepanovic P., Marten J., Zahid S., Chaffin M., Liu Y. (2021). Integrative Analysis of the Plasma Proteome and Polygenic Risk of Cardiometabolic Diseases. Nat. Metab..

[21] Mars N., Kerminen S., Feng Y.-C.A., Kanai M., Läll K., Thomas L.F., Skogholt A.H., Della Briotta Parolo P., FinnGen, Biobank Japan Project (2022). Genome-Wide Risk Prediction of Common Diseases across Ancestries in One Million People. Cell Genom..

[22] Ye Y., Chen X., Han J., Jiang W., Natarajan P., Zhao H. (2021). Interactions Between Enhanced Polygenic Risk Scores and Lifestyle for Cardiovascular Disease, Diabetes, and Lipid Levels. Circ. Genom. Precis. Med..

[23] Tamlander M., Mars N., Pirinen M., Palotie A., Daly M., Riley-Gills B., Jacob H., Paul D., Runz H., John S. (2022). Inte-gration of Questionnaire-Based Risk Factors Improves Polygenic Risk Scores for Human Coronary Heart Disease and Type 2 Diabetes. Commun. Biol..

[24] Khera A.V., Chaffin M., Aragam K.G., Haas M.E., Roselli C., Choi S.H., Natarajan P., Lander E.S., Lubitz S.A., Ellinor P.T. (2018). Genome-Wide Polygenic Scores for Common Diseases Identify Individuals with Risk Equivalent to Monogenic Mutations. Nat. Genet..

[25] Polfus L.M., Darst B.F., Highland H., Sheng X., Ng M.C.Y., Below J.E., Petty L., Bien S., Sim X., Wang W. (2021). Genetic Discovery and Risk Characterization in Type 2 Diabetes across Diverse Populations. HGG Adv..

[26] Sinnott-Armstrong N., Tanigawa Y., Amar D., Mars N., Benner C., Aguirre M., Venkataraman G.R., Wainberg M., Ollila H.M., Kiiskinen T. (2021). Genetics of 35 Blood and Urine Biomarkers in the UK Biobank. Nat. Genet..

[27] Wong C.K., Makalic E., Dite G.S., Whiting L., Murphy N.M., Hopper J.L., Allman R. (2022). Polygenic Risk Scores for Cardiovascular Diseases and Type 2 Diabetes. PLoS ONE.

[28] Wedekind L.E., Mahajan A., Hsueh W.-C., Chen P., Olaiya M.T., Kobes S., Sinha M., Baier L.J., Knowler W.C., McCarthy M.I. (2023). The Utility of a Type 2 Diabetes Polygenic Score in Addition to Clinical Variables for Prediction of Type 2 Diabetes Incidence in Birth, Youth and Adult Cohorts in an Indigenous Study Population. Diabetologia.

[29] Mansour Aly D., Dwivedi O.P., Prasad R.B., Käräjämäki A., Hjort R., Thangam M., Åkerlund M., Mahajan A., Udler M.S., Florez J.C. (2021). Genome-Wide Association Analyses Highlight Etiological Differences Underlying Newly Defined Subtypes of Diabetes. Nat. Genet..

[30] Tanigawa Y., Qian J., Venkataraman G., Justesen J.M., Li R., Tibshirani R., Hastie T., Rivas M.A. (2022). Significant Sparse Polygenic Risk Scores across 813 Traits in UK Biobank. PLoS Genet..

[31] Shoaib M., Ye Q., IglayReger H., Tan M.H., Boehnke M., Burant C.F., Soleimanpour S.A., Gagliano Taliun S.A. (2023). Evaluation of Polygenic Risk Scores to Differentiate between Type 1 and Type 2 Diabetes. Genet. Epidemiol..

[32] Vassy J.L., Hivert M.-F., Porneala B., Dauriz M., Florez J.C., Dupuis J., Siscovick D.S., Fornage M., Rasmussen-Torvik L.J., Bouchard C. (2014). Polygenic Type 2 Diabetes Prediction at the Limit of Common Variant Detection. Diabetes.

[33] Lamri A., Limbachia J., Schulze K., Desai D., Kelly B., de Souza R., Paré G., Lawlor D., Wright J., Anand S. (2022). The Genetic Risk of Gestational Diabetes in South Asian Women. MedRxiv.

[34] Kim Y.J., Moon S., Hwang M.Y., Han S., Jang H.-M., Kong J., Shin D.M., Yoon K., Kim S.M., Lee J.-E. (2022). The Con-tribution of Common and Rare Genetic Variants to Variation in Metabolic Traits in 288,137 East Asians. Nat. Commun..

[35] Pezzilli S., Tohidirad M., Biagini T., Scarale M.G., Alberico F., Mercuri L., Mannino G.C., Garofolo M., Filardi T., Tang Y. (2022). Contribution of Rare Variants in Monogenic Diabetes-Genes to Early-Onset Type 2 Diabetes. Diabetes Metab..

[36] Qi Q., Stilp A.M., Sofer T., Moon J.-Y., Hidalgo B., Szpiro A.A., Wang T., Ng M.C.Y., Guo X., MEta-analysis of type 2 DIabetes in African Americans (MEDIA) Consortium (2017). Genetics of Type 2 Diabetes in U.S. Hispanic/Latino Individuals: Results from the Hispanic Community Health Study/Study of Latinos (HCHS/SOL). Diabetes.

[37] Siddiqui M.K., Anjana R.M., Dawed A.Y., Martoeau C., Srinivasan S., Saravanan J., Madanagopal S.K., Taylor A., Bell S., Veluchamy A. (2022). Correction to: Young-Onset Diabetes in Asian Indians Is Associated with Lower Measured and Genetically Determined Beta Cell Function. Diabetologia.

[38] Kerminen S., Martin A.R., Koskela J., Ruotsalainen S.E., Havulinna A.S., Surakka I., Palotie A., Perola M., Salomaa V., Daly M.J. (2019). Geographic Variation and Bias in the Polygenic Scores of Complex Diseases and Traits in Finland. Am. J. Hum. Genet..

[39] Naret O., Kutalik Z., Hodel F., Xu Z.M., Marques-Vidal P., Fellay J. (2022). Improving Polygenic Prediction with Genetically Inferred Ancestry. Hum. Genet. Genom. Adv..

[40] Koch S., Schmidtke J., Krawczak M., Caliebe A. (2023). Clinical Utility of Polygenic Risk Scores: A Critical 2023 Appraisal. J. Community Genet..

[41] Martin A.R., Kanai M., Kamatani Y., Okada Y., Neale B.M., Daly M.J. (2019). Clinical Use of Current Polygenic Risk Scores May Exacerbate Health Disparities. Nat. Genet..

[42] Choi S.W., García-González J., Ruan Y., Wu H.M., Porras C., Johnson J., Hoggart C.J., O’Reilly P.F., Bipolar Disorder Working group of the Psychiatric Genomics Consortium (2023). PRSet: Pathway-Based Polygenic Risk Score Analyses and Software. PLoS Genet..

[43] Taliun D., Harris D.N., Kessler M.D., Carlson J., Szpiech Z.A., Torres R., Taliun S.A.G., Corvelo A., Gogarten S.M., Kang H.M. (2021). Sequencing of 53,831 Diverse Genomes from the NHLBI TOPMed Program. Nature.

[44] Choi S.W., Mak T.S.-H., O’Reilly P.F. (2020). Tutorial: A Guide to Performing Polygenic Risk Score Analyses. Nat. Protoc..

[45] Collister J.A., Liu X., Clifton L. (2022). Calculating Polygenic Risk Scores (PRS) in UK Biobank: A Practical Guide for Epidemiologists. Front. Genet..

[46] Purcell S., Neale B., Todd-Brown K., Thomas L., Ferreira M.A.R., Bender D., Maller J., Sklar P., de Bakker P.I.W., Daly M.J. (2007). PLINK: A Tool Set for Whole-Genome Association and Population-Based Linkage Analyses. AJHG.

[47] Danecek P., Bonfield J.K., Liddle J., Marshall J., Ohan V., Pollard M.O., Whitwham A., Keane T., McCarthy S.A., Davies R.M. (2021). Twelve Years of SAMtools and BCFtools. GigaScience.

[48] Durbin R.M., Altshuler D., Durbin R.M., Abecasis G.R., Bentley D.R., Chakravarti A., Clark A.G., Collins F.S., De La Vega F.M., Donnelly P. (2010). A Map of Human Genome Variation from Population-Scale Sequencing. Nature.

[49] Koenig Z., Yohannes M.T., Nkambule L.L., Goodrich J.K., Kim H.A., Zhao X., Wilson M.W., Tiao G., Hao S.P., Sahakian N. (2023). A Harmonized Public Resource of Deeply Sequenced Diverse Human Genomes. bioRxiv.

[50] R Core Team (2020). R: A Language and Environment for Statistical Computing.

[51] Robin X., Turck N., Tiberti N., Lisacek F., Sanchez J.-C., Müller M., Siegert S., Doering M., Billings Z. (2023). pROC: Display and Analyze ROC Curves; R Package Version 1.18.5. https://cran.r-project.org/web/packages/pROC/index.html.

[52] Revelle W. (2023). Psych: Procedures for Psychological, Psychometric, and Personality Research; R Package Version 2.3.9. https://CRAN.R-project.org/package=psych.

[53] Chongsuvivatwong V. (2022). Epidemiological Data Display Package; R Package Version 3.5.0.2. https://CRAN.R-project.org/package=epiDisplay.

[54] Fraley C., Raftery A.E., Scrucca L., Murphy T.B., Fop M. (2023). Mclust: Gaussian Mixture Modelling for Model-Based Clustering, Classification, and Density Estimation; R Package Version 6.0.1. https://cran.r-project.org/web/packages/mclust/index.html.

[55] Nakazawa M. (2023). Fmsb: Functions for Medical Statistics Book with Some Demographic Data; R Package Version 0.7.5. https://cran.r-project.org/web/packages/fmsb/index.html.

[56] Ruan Y., Lin Y.-F., Feng Y.-C.A., Chen C.-Y., Lam M., Guo Z., Ahn Y.M., Akiyama K., Arai M., Baek J.H. (2022). Improving polygenic prediction in ancestrally diverse populations. Nat. Genet..

[57] Mak T.S.H., Porsch R.M., Choi S.W., Zhou X., Sham P.C. (2017). Polygenic scores via penalized regression on summary statistics. Genet. Epidemiol..

[58] Loh P.-R., Tucker G., Bulik-Sullivan B.K., Vilhjalmsson B.J., Finucane H.K., Salem R.M., Chasman D.I., Ridker P.M., Neale B.M., Berger B. (2015). Efficient Bayesian mixed model analysis increases association power in large cohorts. Nat. Genet..

[59] Zheng Z., Liu S., Sidorenko J., Yengo L., Turley P., Ani A., Wang R., Nolt I., Snieder H., Lifelines Cohort Study Yang J. (2022). Leveraging functional genomic annotations and genome coverage to improve polygenic prediction of complex traits within and between ancestries. bioRxiv.

