# Supplementary figures and images for: Evaluating the Efficacy of Type 2 Diabetes Polygenic Risk Scores in an Independent European Population
Source: Int J Mol Sci. 2024 Jan 17;25(2):1151. doi: 10.3390/ijms25021151 (PMC10817091; doi:10.3390/ijms25021151)

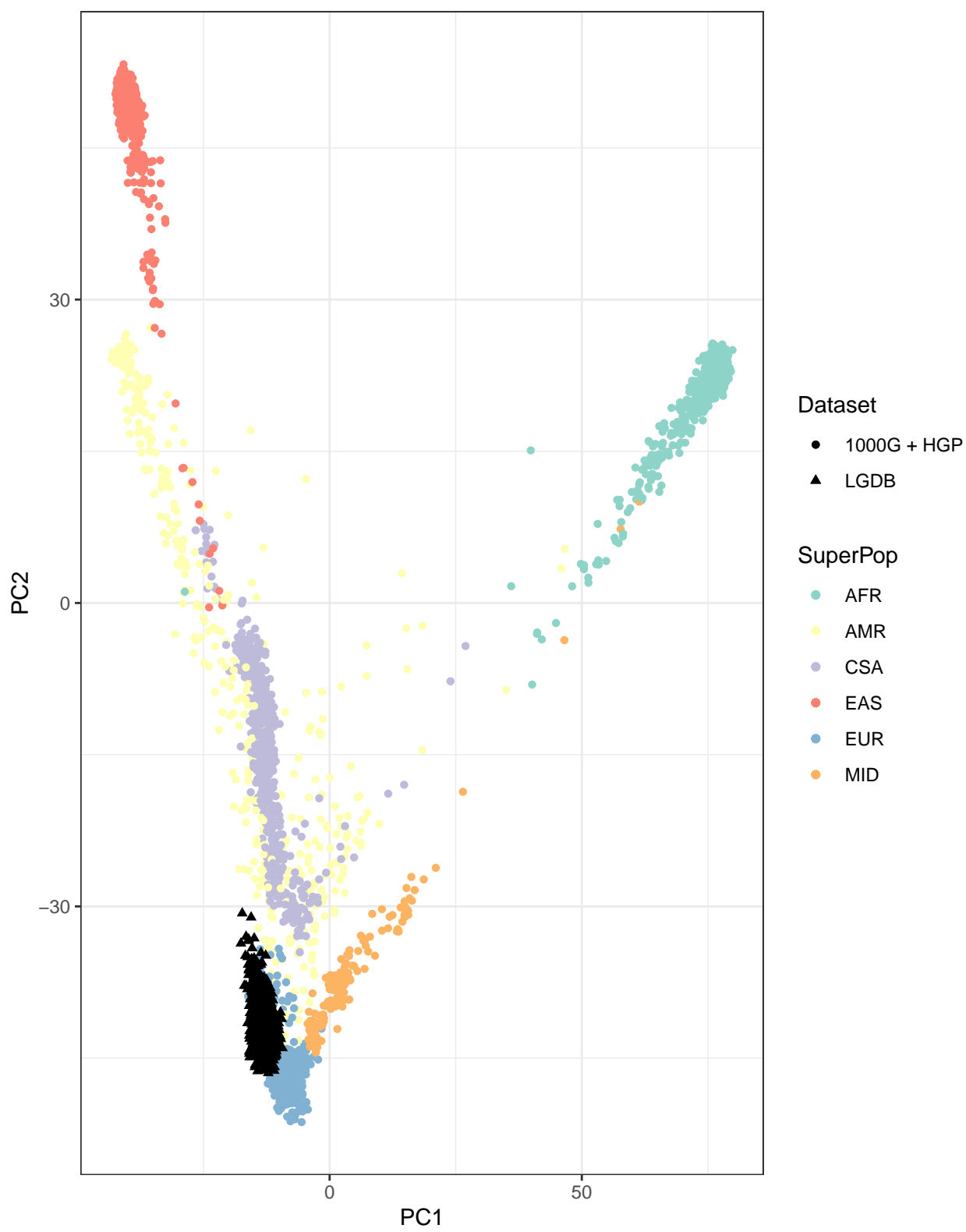

Supplement: Supplementary file 1 [file ijms-25-01151-s001.zip › Figure S1.pdf]

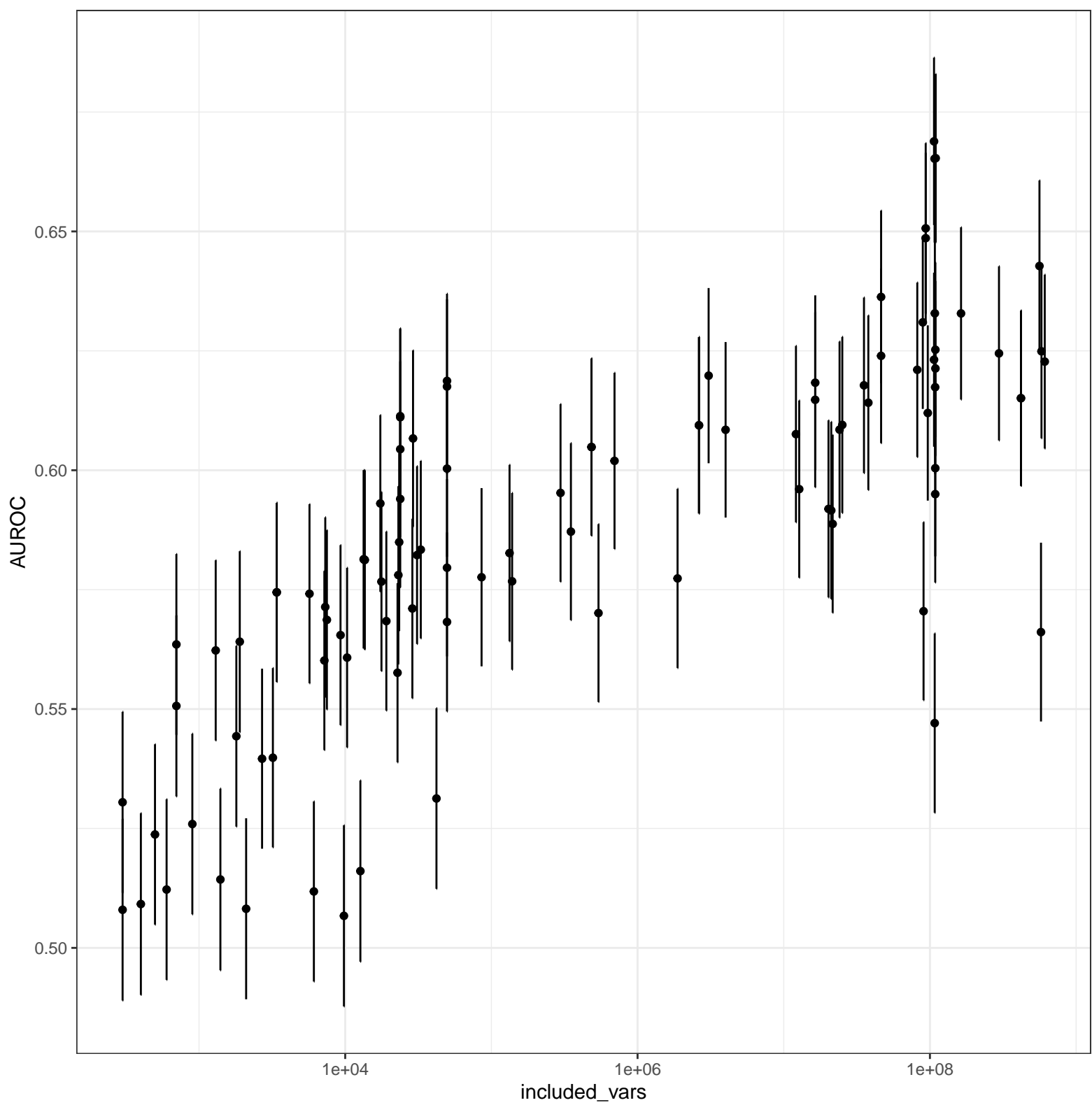

Supplement: Supplementary file 1 [file ijms-25-01151-s001.zip › Figure S2.pdf]

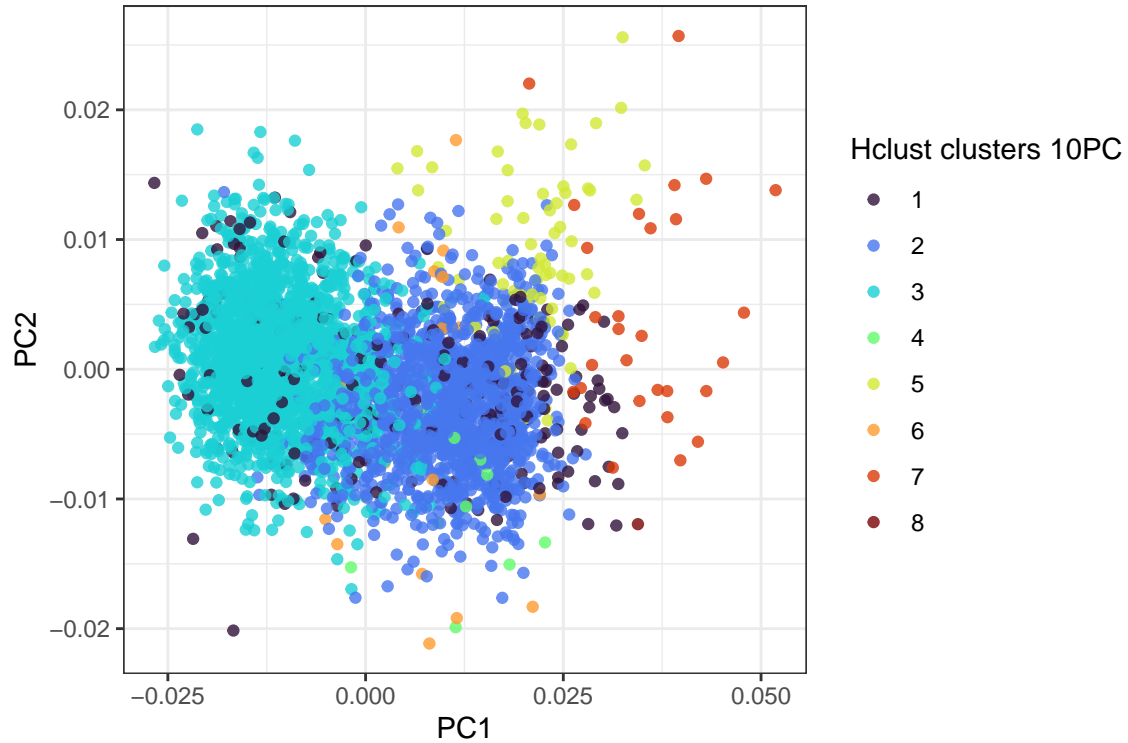

Supplement: Supplementary file 1 [file ijms-25-01151-s001.zip › Figure S3.pdf]

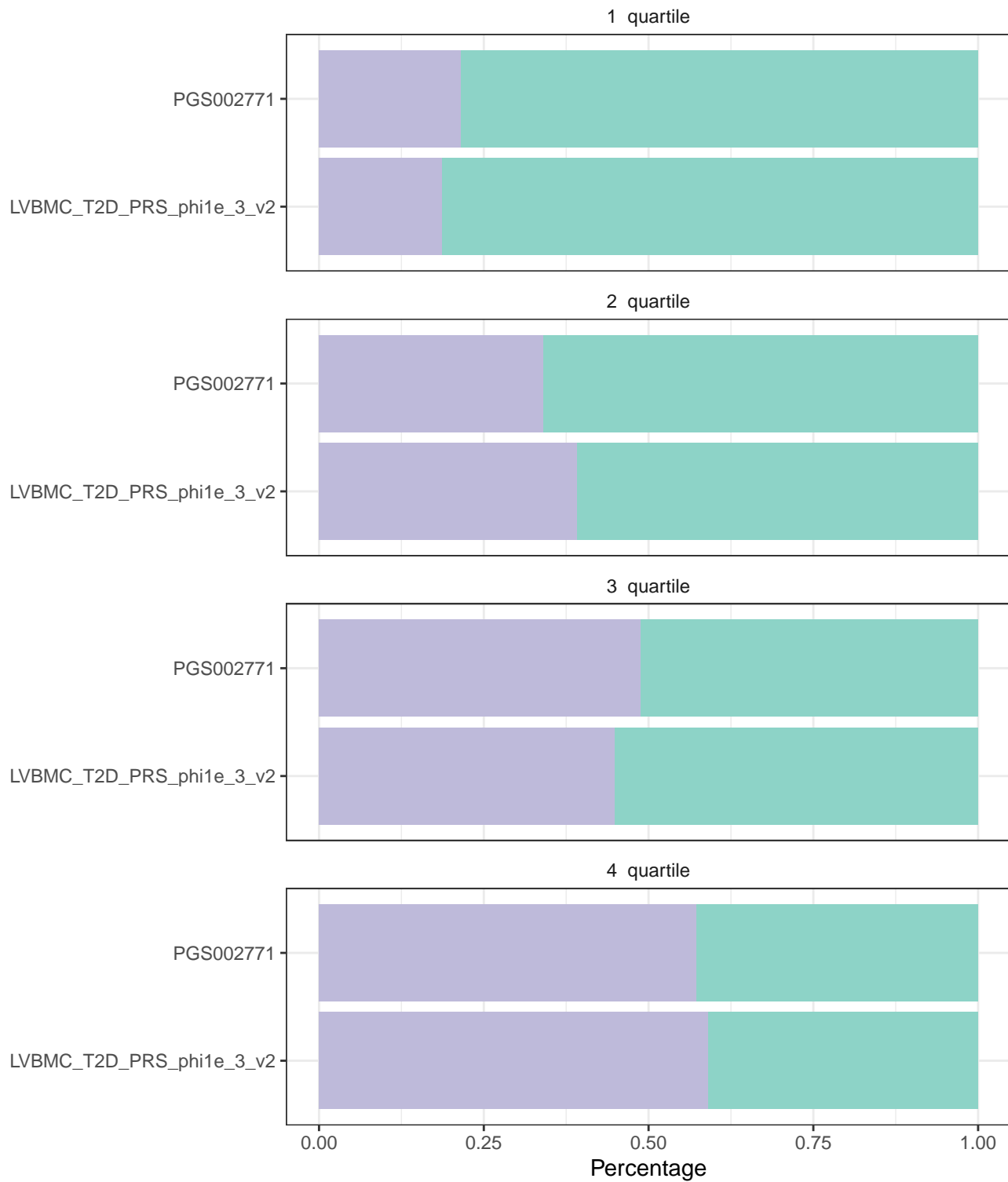

Supplement: Supplementary file 1 [file ijms-25-01151-s001.zip › Figure S4.pdf]
